# Cell‐based high‐throughput screen for small molecule inhibitors of Bax translocation

**DOI:** 10.1111/jcmm.14076

**Published:** 2018-12-13

**Authors:** Kelvin Kai‐Wan Hui, Chesarahmia Dojo Soeandy, Stephano Chang, Frederick S. Vizeacoumar, Thomas Sun, Alessandro Datti, Jeffrey T. Henderson

**Affiliations:** ^1^ Department of Pharmaceutical Sciences, Leslie Dan Faculty of Pharmacy University of Toronto Toronto ON Canada; ^2^ RIKEN Center for Brain Science Wako Japan; ^3^ Department of Pathology and Laboratory Medicine Royal University Hospital, College of Medicine, University of Saskatchewan Saskatoon SK Canada; ^4^ Mount Sinai Hospital, Lunenfeld‐Tanenbaum Research Institute Toronto ON Canada; ^5^ SMART Laboratory for High‐Throughput Screening Programs Mount Sinai Hospital, Network Biology Collaborative Centre Toronto ON Canada; ^6^ Department of Agriculture, Food, and Environmental Sciences University of Perugia Perugia Italy

**Keywords:** apoptosis, bax, cell‐based assay, high‐throughput screen, in vivo, programmed cell death inhibitor, small molecular inhibitor

## Abstract

Aberrant regulation of programmed cell death (PCD) has been tied to an array of human pathologies ranging from cancers to autoimmune disorders to diverse forms of neurodegeneration. Pharmacologic modulation of PCD signalling is therefore of central interest to a number of clinical and biomedical applications. A key component of PCD signalling involves the modulation of pro‐ and anti‐apoptotic Bcl‐2 family members. Among these, Bax translocation represents a critical regulatory phase in PCD. In the present study, we have employed a high‐content high‐throughput screen to identify small molecules which inhibit the cellular process of Bax re‐distribution to the mitochondria following commitment of the cell to die. Screening of 6246 Generally Recognized As Safe compounds from four chemical libraries post‐induction of cisplatin‐mediated PCD resulted in the identification of 18 compounds which significantly reduced levels of Bax translocation. Further examination revealed protective effects via reduction of executioner caspase activity and enhanced mitochondrial function. Consistent with their effects on Bax translocation, these compounds exhibited significant rescue against in vitro and in vivo cisplatin‐induced apoptosis. Altogether, our findings identify a new set of clinically useful small molecules PCD inhibitors and highlight the role which cAMP plays in regulating Bax‐mediated PCD.

## 1 INTRODUCTION

Dysregulation of programmed cell death (PCD) in mammals has been associated with cellular abnormalities ranging from autoimmunity to neurodegeneration and cancer.^1^ Indeed, many of the most frequent gene mutations in human cancers directly or indirectly regulate PCD.^2^ Molecular interactions governing cell death signalling have therefore generated considerable interest as potential targets for pharmacologic PCD modulation. Studies performed over the past three decades have identified major pathways governing PCD initiation and execution.^3^ The first of these termed the intrinsic pathway regulates cellular responses to genotoxic stress, growth factor withdrawal, calcium influx and disruption of the cytoskeletal network, while the second (extrinsic) pathway is initiated following the extracellular binding of death receptor ligands as Fas ligand, tumour necrosis factor‐α (TNF‐α) and TNF‐related apoptosis‐inducing ligand (TRAIL).^1^

Although the intrinsic and extrinsic PCD pathways are regulated by distinct sets of upstream stimuli, they converge at the level of the mitochondria where interactions between pro‐ and anti‐apoptotic Bcl‐2 family members regulate mitochondrial outer membrane permeability (MOMP).^4^ Once induced to their active conformation by interactions with BH3‐only proteins, Bax and Bak undergo oligomerization and insertion into the mitochondrial outer membrane, promoting release of the mitochondrial second messengers such as cytochrome c, apoptosis‐inducing factor (AIF), second mitochondria‐derived activator of caspases (Smac)/DIABLO, and endonuclease G from the mitochondrial intermembranous space, modifies the mitochondrial matrix, and accelerates mitochondrial fission, thereby reducing levels of oxidative phosphorylation.^5^ Released cytochrome c and Smac/DIABLO subsequently act to promote downstream caspase activation, while AIF and endonuclease G spawn caspase‐independent modes of cell death.^3^ Bax/Bak activity is opposed by the actions of anti‐apoptotic Bcl‐2 proteins such as Bcl‐2, Bcl‐xL, Bcl‐w and Mcl‐1 through either direct suppression of oligomerization or interaction with a unique subgroup of BH3‐only proteins.^6^, ^7^, ^8^ The critical role of Bax and Bak in PCD regulation is highlighted by studies of Bax/Bak double knockouts in which cells exhibit substantial protection from a wide array of PCD inducers.^9^ Further analysis demonstrates that a majority of cell types depend principally upon Bax to execute MOMP.^10^ Thus in a wide array of tissues, Bax function as the primary gatekeeper of mitochondrial MOMP, and ultimately PCD progression.

Genetic and pharmacologic manipulation of Bcl‐2 protein‐protein interactions in vivo has demonstrated that processes which inhibit Bax oligomerization result in a substantial enhancement of neuronal survival following acute or chronic neural injury.^11^, ^12^ Consistent with this, peptides derived from two proteins known to interact with Bax (Ku70 and Bax inhibitor‐1) have been shown to exhibit some ability to suppress Bax activation and in turn apoptotic cell death.^13^, ^14^ However, the administration of such agents exhibit significant clinical challenges due to their low bioavailability and stability in vivo. The possibility of identifying small molecule Bcl‐2‐family modifiers is suggested from several studies aimed at *enhancing* apoptotic response through the inhibition of anti‐apoptotic Bcl‐2 family members.^15^ As a result, the small molecules ABT‐737 and derivative ABT‐263 were identified to inhibit the activity of Bcl‐2, Bcl‐xL and Bcl‐w interactions at nanomolar concentrations and show promise in early clinical trials.^16^ Conversely, the ability to inhibit the pro‐apoptotic functions of Bax is similarly highly desirable clinically in the context of neural preservation following central nervous system (CNS) damage such as stroke and spinal cord injury.

Previous studies have demonstrated the feasibility of using fully functional enhanced green fluorescent protein (EGFP)‐Bax and other PCD fusion proteins as real‐time detectors of apoptotic progression.^17^ Given that the translocation of Bax from the cytoplasm to the mitochondria serves as an early and readily detectable indicator of functional apoptotic progression, we engineered an EGFP‐Bax fusion protein to monitor the relative levels of Bax translocation in a temporal manner. Cell lines stably expressing this fusion were then examined in the context of a high‐content, high‐throughput chemical library screen following commitment of the cells to die in order to identify potential small molecule Bax inhibitors. From our screen of over 6000 compounds, we identified two Generally Recognized As Safe (GRAS) compounds with similar mechanism of action which promoted substantial reductions in Bax translocation following cisplatin‐mediated PCD stimulation. Further validation of these agents in vivo demonstrated their ability to suppress apoptotic PCD in the murine brain following cisplatin challenge. Taken together, the results demonstrate that modulation of cAMP signalling using several novel small molecule inhibitors can be used to alter levels of Bax activation and apoptotic cell death in the mammalian CNS.

## 2 MATERIALS AND METHODS

### 2.1 EGFP‐Bax expression vector

The mouse Bax cDNA was PCR amplified from IMAGE clone 3968903 and cloned into mammalian expression vector pEGFP‐C1 (Clontech Laboratories, Inc.) via BglII and EcoRI sites. All clones were sequence verified.

### 2.2 Cell culture and transfection

Certified Chinese Hamster Ovary (CHO) cells were maintained at 37°C, 6% CO_2_ in Dulbecco's Modified Eagle Medium (DMEM, 25 mmol/L HEPES) supplemented with 10% heat‐inactivated foetal bovine serum (Invitrogen Corp., 12483020), 2 mmol/L glutamine and 1% antibiotics (penicillin and streptomycin) (Invitrogen Corp., 10378016). For generation of stable cell lines, EGFP‐Bax expression vector was linearized at the MluI site and transfected into CHO cells by standard calcium phosphate‐mediated method. Geneticin (G418, Sigma‐Aldrich Co., G8168) was utilized for selection at a concentration of 0.8 mg/mL with G418 media changed every 3 days for a period of 2 weeks prior to cloning individual sub‐colonies in 24‐well plates. Independent isolates were cloned and analysed for levels of EGFP‐Bax expression by fluorescent microscopy. Optimal EGFP‐Bax‐expressing lines were expanded through three serial passages, re‐tested and frozen for long‐term cryostorage until use.

### 2.3 Analysis of Bax translocation

To determine the levels of cellular Bax translocation in each cell, Cellomics ArrayScan HCS images were analysed in a blinded manner using a modified Spot Detection algorithm. Briefly, cells registering a detectable EGFP profile within a cellular domain containing a contiguous DAPI profile (XF53 dichroic filter, Omega Optical; ex. 475 nm, em. 525; ex. 365, em. 525; respectively) were scored as a function of their EGFP distribution and signal intensity (Supporting Information Figure S1). Cells undergoing PCD demonstrated redistribution of EGFP from the cell cytoplasm to punctuate localizations adjacent to the (DAPI^+^) cell nucleus. Under these conditions, cells exhibit a dramatic rise in EGFP pixel intensity. Based on the fluorescence intensity in the EGFP channel, the modified Spot Detection algorithm placed red dots in the cell cytoplasm corresponding to areas with relocalized Bax. Specifically, a cell was considered to have undergone Bax translocation if the algorithm detected greater than 20 spots. Cells with low EGFP fluorescent intensity regardless of localization or partially imaged in field (ie nuclei on the edges of the images were excluded from analysis by the algorithm). For each well, results of a minimum of 250 separate cellular profiles were examined from 20‐30 independent image fields. For each compound tested, cellular results were recorded as percent translocation per total cellular nuclear profiles. Translocation levels identified as statistically significant “hits” were determined by the above criteria and program parameters informed by machine learning based on experimental test sets comprising >2000 vehicle or cisplatin‐treated cells from ≥600 individual treatment wells. Results were reported as a relative percentage of cellular population exhibiting translocation. As additional controls, 16 vehicle and 16 cisplatin‐treated wells were included in each 384‐well plate as plate‐internal negative and positive controls. For cisplatin‐treated controls, observed levels of translocation were typically 68%‐71% of native cell population in fields assessed. This level of cellular translocation was set as 100% in normalized translocation assays and results deviating by ≥4σ from the normalized distribution were flagged as potential drug modifier. Compounds identified in this manner were re‐tested in order to verify the results obtained.

### 2.4 High‐throughput screening for small molecule inhibitors of Bax translocation

Stably transfected EGFP‐Bax CHO cells were plated at a density of 4000 cells per well in 384‐well plates. Following overnight incubation, cells were treated with 50 μg/mL cisplatin (Sigma‐Aldrich Co., P4394). At the indicated time, cisplatin‐containing cell culture media was then removed and replaced with fresh media containing vehicle or chemical library constituents to a final concentration of 5 μmol/L (200 nL of 1 mmol/L stock in a total well volume of 40 μL). Cells were incubated further for 24 hours and fixed in 4% paraformaldehyde in phosphate‐buffered saline (PBS, pH 7.4). Hoechst‐33258 (Sigma‐Aldrich Co., B2883) was added to a final concentration of 2 μg/mL for 10 minutes to provide fluorescent nuclear detection. Cells were then washed with PBS, and tiled arrays of EGFP‐Bax CHO cells from each well were captured using a Cellomics ArrayScan HCS Reader (Thermo Fisher Scientific, Inc.). For each 384‐well plate, results from 16 independent controls wells were normalized as a baseline prior to plotting small molecule data.

### 2.5 Executioner caspase activity

Executioner caspase (DEVDase) activity was measured using a SensoLyte Homogeneous Rh110 Caspase‐3/7 Assay Kit (Anaspec, Inc., 71114) according to the manufacturer's instructions. Briefly, 1.0 × 10^4^ cells were plated into each well of a 96‐well plate for overnight incubation and subjected to the experimental treatments indicated. At the designated time‐points, 33 μL of freshly prepared fluorogenic caspase substrate (DEVD‐Rho110) was added to wells containing 100 μL cell culture media and incubated at room temperature for 18 hours. Following incubation, fluorescence was determined using a microplate fluorometer (ex. 496 nm, em. 520 nm; Molecular Devices, SpectraMax M2).

### 2.6 Trypan blue exclusion

For trypan blue exclusion assay, 2.5 × 10^4^ cells were plated into each well of a 24‐well plate for overnight incubation and subjected to the experimental treatments indicated. At the designated time‐points, cells were detached using 0.25% trypsin in saline/ethylenediaminetetraacetic acid and the solution recombined with the culture media collected from the same well (as it may contain potential floating dead cells). Samples were pelleted by centrifugation at 800× *g* for 5 minutes and the pellets re‐suspended in 100 μL cell culture media and diluted 1:1 with 0.4% trypan blue (Invitrogen Corp., 15250061). Counts were performed with a haemocytometer with cell viability reported as the percentage of trypan blue excluding cells divided by total cell number.

### 2.7 Lactate dehydrogenase assay

Lactate dehydrogenase (LDH) was measured using CytoTox‐ONE Homogenous Membrane Integrity Assay (Promega Corp., G7890) according to the manufacturer's instructions. Briefly, 4.0 × 10^4 ^cells were plated into each well of a 96‐well plates for overnight incubation and subjected to the experimental treatments indicated. At the designated time‐points, samples were equilibrated to room temperature and 100 μL freshly prepared resazurin containing solution was added to individual wells holding 100 μL cell culture media. Samples were incubated at room temperature for 10 minutes and fluorescent measurements were made using a fluorescence microplate reader (excitation 560 nm, emission 590 nm; Molecular Devices, Inc., SpectraMax M2).

### 2.8 MTT assay

For 3‐(4,5‐Dimethylthiazol‐2‐yl)‐2,5‐diphenyltetrazolium bromide (MTT) reduction assays, 1.0 × 10^5^ cells were plated onto 96‐well plates for overnight incubation and subjected to the experimental treatments indicated. At the designated time‐points, 25 μL of freshly prepared MTT solution (5 mg/mL in PBS) was added to individual wells (containing 100 μL cell culture media) and incubated at 37°C for 2 hours. Following incubation, 100 μL of MTT extraction buffer (20% SDS [w/v], 50% dimethyl formamide in deionized distilled water, pH 4.7) was added to each well. Following overnight incubation at room temperature in a humidified chamber, colorimetric measurements were recorded using a multi‐well microplate reader (absorbance 570 nm, reference 690 nm; Molecular Devices, Inc., SpectraMax M2).

### 2.9 In vivo cisplatin assay

Mice were injected subcutaneously on postnatal day 2 with freshly prepared cisplatin (7 mg/kg) or saline control at a volume of 10 μL/g body weight. Hit compounds or vehicle (ethanol) were then injected 1 hour following cisplatin treatment. For some animals, the caspase‐3/7 inhibitor z‐DEVD‐fmk (Bachem Americas, Inc., 4027402) was injected 30 minutes prior to cisplatin treatment as a positive control. For these experiments, forskolin, rolipram and DEVD were first dissolved in 100% anhydrous ethanol, then diluted in 0.9% saline to a final concentration of 4 mmol/L (25% ethanol injected at 10 µL/g body weight). Animals were killed at 24 hours following cisplatin treatment with the cerebella carefully removed and fixed in 4% paraformaldehyde with gentle agitation. Cerebella were then rinsed and prepared for paraffin wax sectioning. For each animal, a series of 7‐μm parasagittal sections through the vermis were prepared 150 μm lateral to the midline at intervals of 100 μm, six sections for each series. Slides were dewaxed and prepared for TUNEL staining as described previously.^18^ The numbers of TUNEL^+ ^cells were determined as a function of external germinal layer (EGL) area quantified in lobes VI and VIII of the cerebellum. All procedures were in carried out in accordance with University of Toronto Animal Protocols.

### 2.10 Statistical analyses

Statistical tests including Student's *t* test and one‐way anova with Bonferroni's multiple comparison test were performed with Microsoft Excel or Prism GraphPad. Data are presented as mean ± SEM. Results were considered statistically significant if *P* < 0.05.

## 3 RESULTS

### 3.1 Analysis of EGFP‐Bax expressing cells

**Figure 1:**
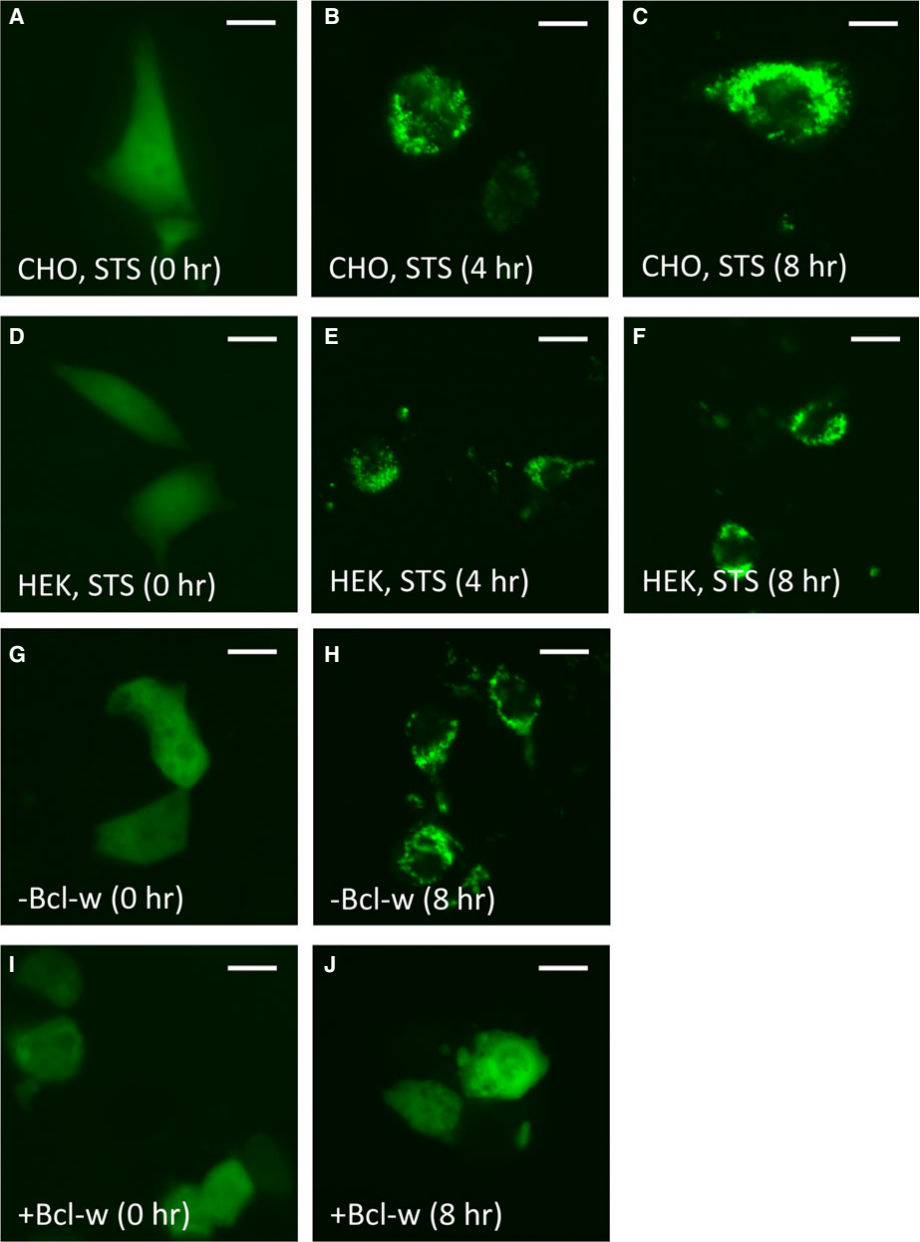
Time‐dependent and Bcl‐w‐suppressible translocation of EGFP‐Bax following programmed cell death (PCD) initiation in Certified Chinese Hamster Ovary (CHO) and human embryonic kidney (HEK) 293T cells. (A‐F) Following transfection with enhanced green fluorescent protein (EGFP)‐Bax (48 h), CHO and HEK 293T cells were stimulated with 2 μM staurosporine or UVC irradiation (not shown) to initiate PCD. Subcellular localization of EGFP‐Bax was then examined at 0, 4 and 8 h following treatment. EGFP‐Bax was initially distributed homogeneously throughout the cell cytoplasm prior to PCD stimulation in CHO (A) and HEK 293T (D) cells. As observed at 4 (B, E) and 8 h (C, F) following staurosporine treatment, both CHO and HEK 293T cells exhibited punctate re‐localization of EGFP‐Bax to perinuclear regions of the cell. (G‐J) The ability of Bcl‐2 family proteins to functionally suppress EGFP‐Bax translocation was examined using Bcl‐w. Forty‐eight hours following transfection in HEK 293T cells, 2 μM staurosporine was used to initiate PCD. No difference in initial cytoplasmic distribution of EGFP‐Bax was observed prior to staurosporine treatment regardless of Bcl‐w status (G, I). At 8 h following staurosporine treatment, substantially fewer cells co‐transfected with Bcl‐w exhibited a punctate redistribution of EGFP‐Bax compared to cells transfected with EGFP‐Bax alone (H, J); scale bar: 5 μm

In order to monitor Bax translocation in real time following PCD stimulation, we first constructed an expression vector producing full‐length Bax fused with EGFP at its N‐terminus. Transient and stable transfection of this EGFP‐Bax construct into CHO, human embryonic kidney (HEK 293T) cells, or primary murine fibroblasts confirmed correct localization and translocation of the fusion protein following PCD activation. For these studies, PCD was stimulated using staurosporine or ultraviolet C (UVC) irradiation, two well‐characterized apoptotic inducers.^19^ As shown in Figure 1A and D, EGFP‐Bax is homogeneously distributed within the cell cytoplasm prior to PCD stimulation and expression of this construct does not by itself trigger mitochondrial translocation or apoptosis. In contrast, EGFP‐Bax re‐distributed to discreet cellular puncta in a time‐dependent manner following staurosporine treatment (Figure 1B‐C, 1‐F).

Bax activity has been shown to be directly regulable by both pro‐apoptotic BH3 activators and anti‐apoptotic Bcl‐2 family members such as Bcl‐w.^7^, ^8^ We, therefore, assessed whether the translocation of this EGFP‐Bax fusion protein could be functionally inhibited by expression of Bcl‐w. To test this, Bcl‐w was co‐transfected with EGFP‐Bax in HEK 293T cells and PCD induced using staurosporine 48 hours following transfection. Cells expressing Bcl‐w (Figure 1I‐J) exhibited substantially lower levels of EGFP‐Bax translocation compared to EGFP‐Bax alone (Figure 1G‐H) at 8 hours following staurosporine induction. Thus, EGFP‐Bax is both functionally sensitive to Bcl‐w inhibition and translocates in a manner similar to that seen for endogenous Bax.

**Figure 2:**
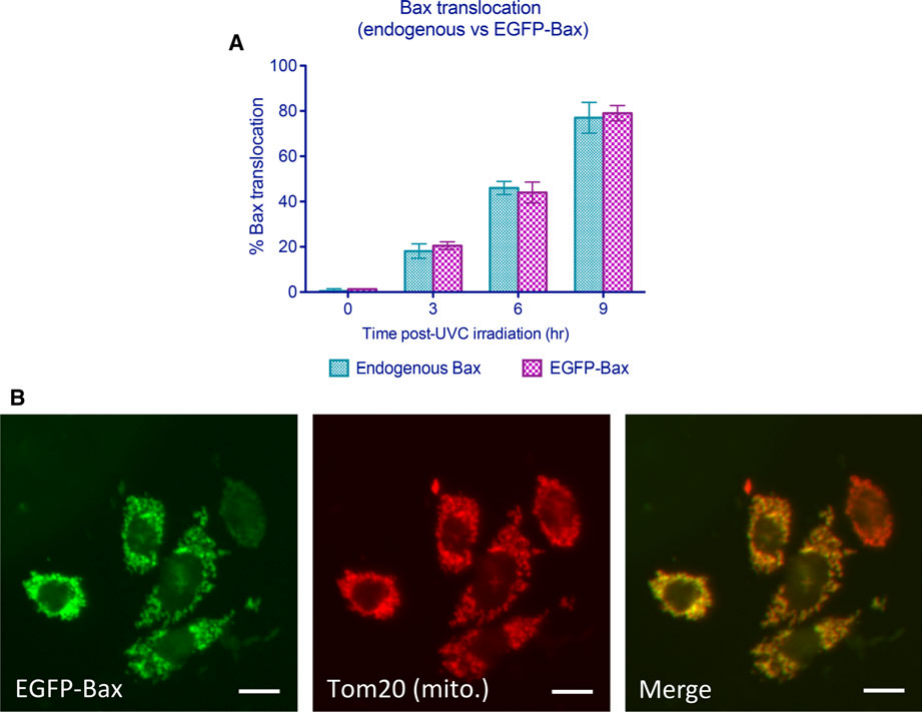
Enhanced green fluorescent protein (EGFP)‐Bax exhibits similar spatial and temporal redistribution as endogenous Bax. (A) Temporal pattern of Bax translocation for EGFP‐Bax and Bax following staurosporine‐mediated induction of programmed cell death. Relative Bax translocation at 0, 3, 6 and 9 h following ultraviolet C (UVC) treatment. Data shown are means ± SEM for experiments performed in triplicates in which ≥100 cells per test condition were examined for each group. No significant differences were observed between endogenous Bax and EGFP‐Bax. (B) In order to determine the fidelity of translocation for EGFP‐Bax, the subcellular localization of EGFP‐Bax was compared in cells to that seen for mitochondrial marker Tom20 as determined by immunofluorescence; scale bar: 5 μm

Based upon these studies, 40 lines of stably transfected EGFP‐Bax CHO cells were constructed. Of these, clones were selected based on criteria such as the consistency of their EGFP‐Bax expression and expression comparable to endogenous Bax. Prior to using EGFP‐Bax CHO cells for screening purposes, we compared the translocation kinetics of these cells to non‐transfected CHO cells at various times following UVC irradiation through direct fluorescence and using anti‐Bax antisera. As shown in Figure 2A, percentages of CHO cells undergoing Bax translocation were similar between groups at each time‐point, demonstrating that Bax translocation following PCD stimulation is not significantly altered between EGFP‐Bax and endogenous Bax. In addition, as shown in Figure 2B, we confirmed proper translocation of EGFP‐Bax to the mitochondria. The subcellular localization of EGFP‐Bax was determined 6 hours following UVC irradiation as a function of Tom20, a well‐characterized component of the translocase outer membrane complex of the mitochondria. Altogether, EGFP‐Bax undergoes appropriate translocation following PCD induction with no significant difference in Bax translocation observed between non‐transfected CHO cells and cells expressing EGFP‐Bax.

### 3.2 Induction of Bax translocation by cisplatin‐mediated cell death

**Figure 3:**
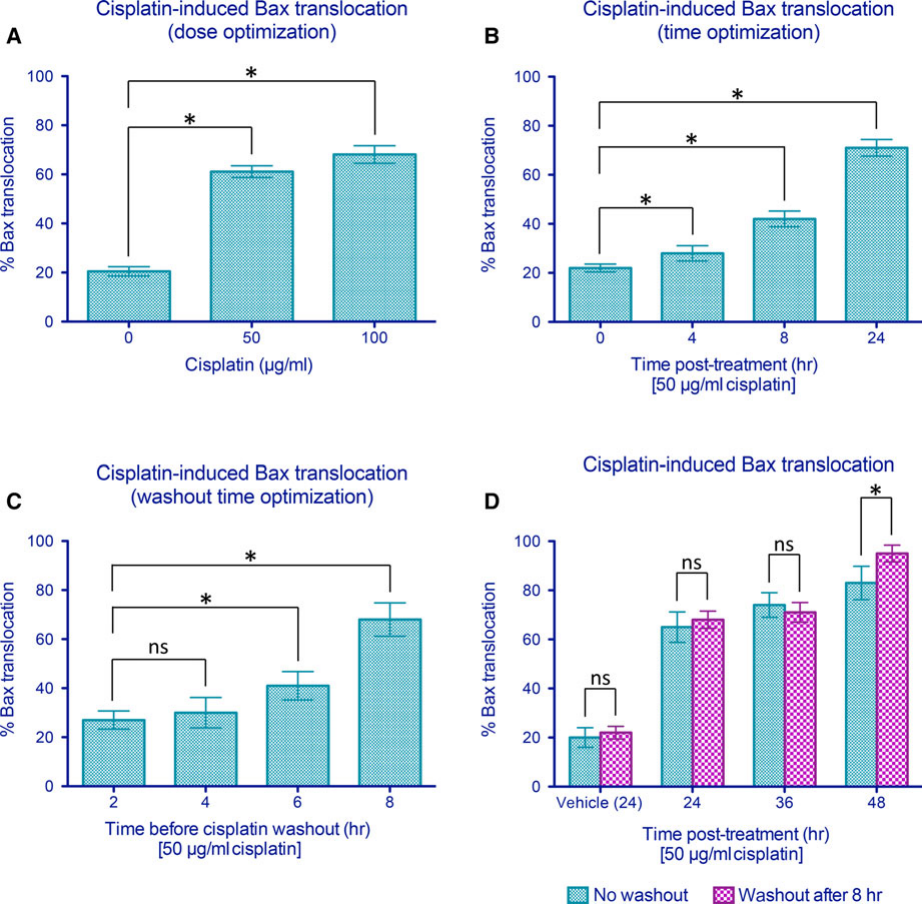
Parameter optimization for cisplatin‐mediated Bax translocation in CHO cells. (A) Cisplatin treatment induces a dose‐dependent enhancement in Bax translocation as seen at 24 h. (B) Significant Bax translocation was observed at 8 and 24 h after treatment with 50 μg/mL cisplatin. (C) Cisplatin (50 μg/mL) was added and removed at different time‐points to determine the minimal treatment duration to elicit significant Bax translocation before washout. Bax translocation was assessed at 24 h after the initiation of cisplatin treatment. (D) Levels of enhanced green fluorescent protein (EGFP)‐Bax translocation sustained following an 8‐hour exposure to cisplatin as assessed after 24, 36 and 48 h. Data shown are means ± SEM from six independent replicates in which *n* ≥ 250 cells per well were examined for each treatment group. * indicates statistical significance at *P* < 0.05 by Student's *t *test between the indicated treatment groups. ns indicates not significant statistically

In order to generate a practical and reproducible PCD model amenable for high‐throughput screening, we examined several chemical inducers of PCD for uniformity of response. Although analyses with staurosporine indicated that this serine/threonine kinase inhibitor induced substantial levels of Bax translocation, it was not ideal for high‐throughput screening due to its notable effects in altering cell morphology which impaired automated cell detection and analysis of EGFP signal distribution. After analysing several chemical agents, cisplatin was eventually selected to induce p53‐dependent PCD. Cisplatin addition to cell cultures resulted in 61% and 68% percent of cells exhibiting Bax translocation at 50 or 100 μg/mL cisplatin, respectively, following 24 hours of treatment (Figure 3A; 96‐well assay format). Bax translocation was observed in a time‐dependent manner as 28%, 42% and 71% of cells following 4, 8 and 24 hours of cisplatin exposure had translocated, respectively (Figure 3B; 384‐well format). In order to determine the effects of a given exposure of cisplatin has on the ultimate induction of Bax translocation, we examined the response to various periods of cisplatin exposure followed by washout and examination at 24 hours. As shown in Figure 3C, levels of Bax translocation continued to rise until approaching levels seen at 24 hours of cisplatin exposure following 8 hours of treatment (384‐well format). Thus, at 50 μg/mL, 8 hours of cisplatin exposure appeared to induce maximal Bax translocation with little additional gain from continued treatment. These data also revealed an important time‐dependent aspect to the commitment of Bax translocation. Incubating cells with cisplatin for 4 hours resulted in 28% of cells exhibiting Bax translocation during this period. Washout of cisplatin at this point did not result in additional increase in Bax translocation when examined at 24 hours (30%). In contrast, exposure of cells to cisplatin for 8 hours resulted in 42% of cells exhibiting Bax translocation, and rising to 68% when examined at 24 hours. Therefore, 8 hours was chosen as the drug addition time‐point for all subsequent analyses as it represented a time at which commitment to the cisplatin‐dependent cell death programme had unequivocally been initiated but where Bax translocation had not yet occurred within the majority of cells committed to die. The stringent nature of this test ensured that any inhibitors of Bax translocation so obtained would likely not act on early commitment steps to PCD.

We further tested whether Bax translocation could be enhanced by extending the analysis periods to create an even more robust assay. However, as shown in Figure 3D, extending the analysis period to 36 or 48 hours following washout at 8 hours had little effect on further enhancement of Bax translocation. An exception to this was the 48 hours “no washout” condition which was not selected as the standard assay because (a) this period of cisplatin exposure resulted in a substantial reduction in the levels of surviving control cells for analysis, (b) cisplatin washout prior to drug addition removed cisplatin‐drug interactions as a potential cause of effects observed and (c) 48 hour exposure time cut assay speed in half with little obvious benefit in terms of greater sensitivity.

### 3.3 Analysis of intra‐ and inter‐plate variability

**Figure 4:**
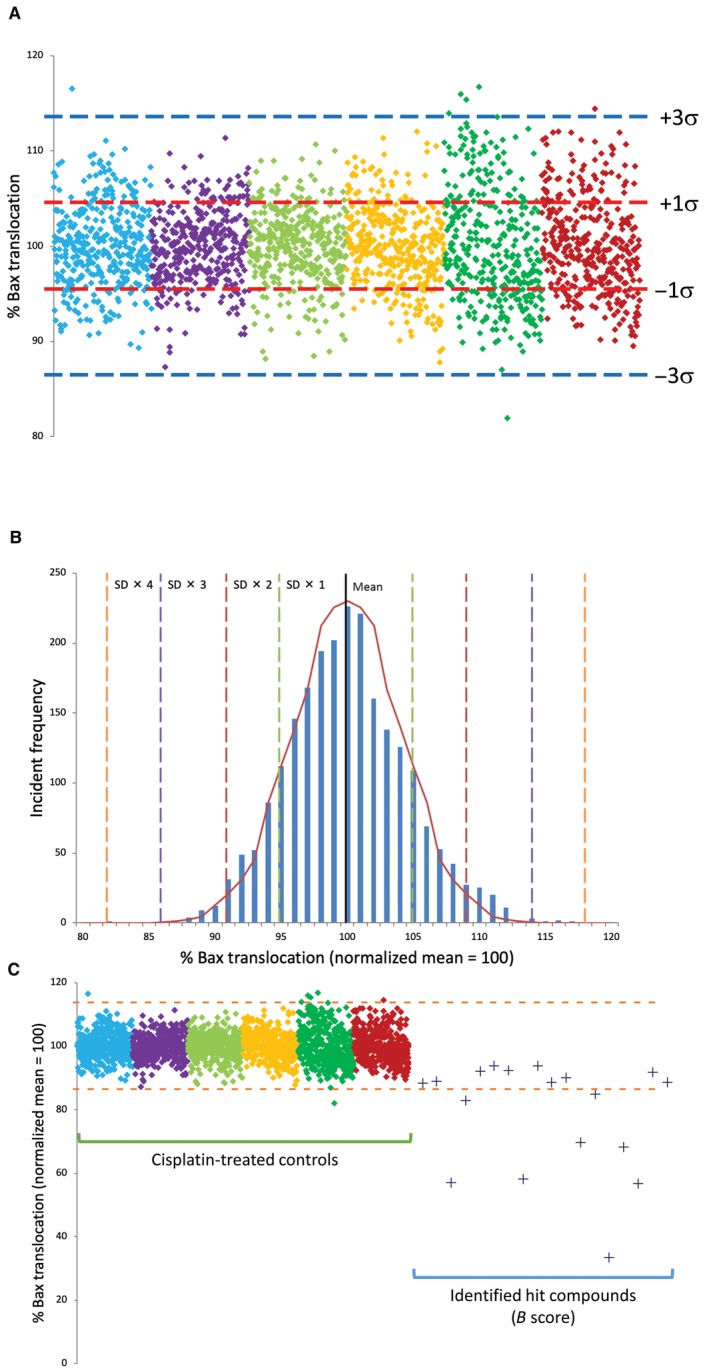
Intra‐ and inter‐plate variability of 384‐well assay for automated enhanced green fluorescent protein (EGFP)‐Bax translocation and identification of small molecule inhibitors of Bax translocation. Assay variability was assessed by examining cisplatin‐induced EGFP‐Bax translocation in six 384‐well plates (2304 wells) following cisplatin treatment. For each well, *n* ≥ 250 cells were examined. (A) Data points represent the level of observed EGFP‐Bax translocation as determined in single wells. Data collected from each plate are represented by different colours normalized to 100% mean. Red and blue and dotted lines represent mean ± 1 and 3 SDs, respectively. (B) Frequency distribution of control data presented in (A) demonstrating normalized distribution data. For control cisplatin data, 99.78% of values fall within 3σ of the normalized mean. Dotted lines represent successive ±SDs away from the normalized mean. Graph line represents idealized normal distribution for this dataset. (C) High‐throughput screening of four chemical libraries (6246 GRAS compounds) identified eight targets who response differed by >3σ from the cisplatin controls dataset. These were selected for secondary screening together with 10 compounds whose B score suggested them as possible hits in comparison to controls. Control data from (A) are shown for reference. For each well, *n* ≥ 250 cells were examined. Blue dotted lines represent the mean ± 3 SD

One of the most critical features of high‐throughput single‐pass assay screens is the variability observed for both inter‐ and intra‐plate assay measures. Such features define both the ultimate limits of assay sensitivity and the likelihood of false‐positive and false‐negative readouts. As a result, estimates of intra‐ and inter‐plate variability were determined in order to assess the relative sensitivity of the assay constructed. Replicates of stable EGFP‐Bax CHO cells were plated onto six 384‐well plates and subjected to PCD stimulation under the defined condition of 50 μg/mL cisplatin for 8 hours. As indicated in Figure 4A, analysis of Bax translocation based upon >250 cells for each of 2304 wells at 24 hours demonstrated a normal distribution with 78.2% of control Bax translocations lying within one SD of the mean. Aggregate dataset exhibited a SD of 4.53. Of 2304 control assays, five exhibited Bax translocation levels greater than three SDs from the mean (0.2%) highlighting the robustness of the assay conditions implemented. These data are represented as a function of σ in Figure 4B.

### 3.4 Screening of small molecule inhibitors of Bax translocation

**Figure 5:**
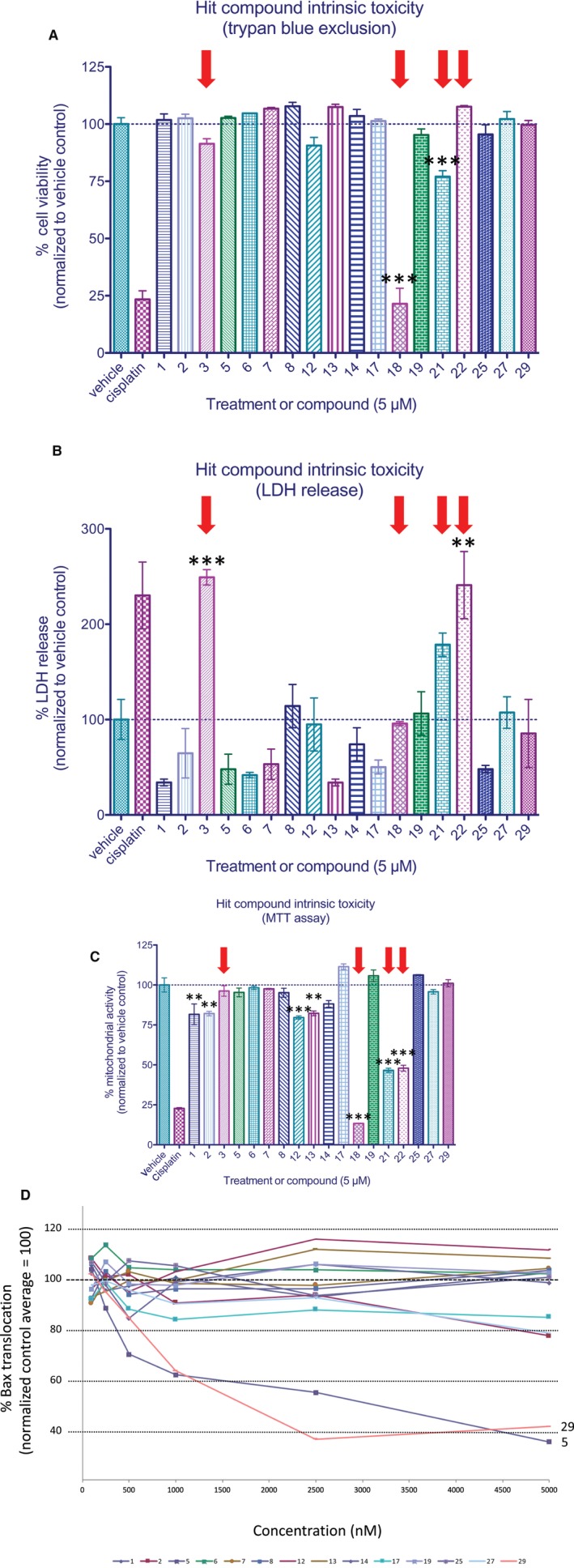
Analysis of intrinsic toxicity and dose‐response of identified compounds. The intrinsic toxicity of identified hits was evaluated using three distinct cell viability assays. Cells were incubated for 24 h in 5 μmol/L of compound prior to analysis. (A) Viability as a function of trypan blue exclusion. (B) Analysis of lactate dehydrogenase release. (C) Analysis of mitochondrial activity as assessed via MTT reduction. Red arrows indicate compounds which exhibited features of intrinsic toxicity and were therefore excluded from subsequent analyses. Data represent means ± SEM from four independent replicates for all treatment groups. For trypan blue exclusion experiments, *n* ≥ 100 cells per well were examined for all treatment groups. (D) Identified compounds were examined at concentrations ranging from 0.1‐5 μmol/L. Compounds 5 and 29 were observed to be significantly more active in inhibiting Bax translocation than other hit compounds. Data represent normalized means ± SEM of four independent replicates in which n ≥ 250 cells per well were examined for all treatment groups. ** and *** indicate statistical significance at *P* < 0.01 and *P* < 0.001 by one‐way anova and Bonferroni's multiple comparison test between the indicated treatment groups

Screening was performed with of four separate chemical libraries (LOPAC, NIH Clinical Collection, Prestwick, and Spectrum) containing distinct chemical entities. Ultimately, 6246 compounds were examined resulting in the identification of 18 compounds indicating significant reductions in Bax translocation compared to cisplatin‐treated controls by B score analysis^20^ (Figure 4C). Based upon the control data, it would be predicted that an average of 2.17 false positives ≥3σ would be observed per 1000 assays, or 13.5 hits (false positives) for the sample set examined. However, six of these compounds demonstrate Bax translocation levels which lay >4σ away from the mean (>81.9%, predicted false positive rate: 1/15787 assays). All 18 compounds were investigated to determine whether the results obtained reflected an inhibition of cell death or were a result of a form of intrinsic cellular toxicity. As shown in Figure 5A‐C, three measures of cellular function were examined: molecular exclusion of trypan blue, lactate dehydrogenase release and reduction of MTT to its insoluble formazan. Cells were treated for 24 hours with the indicated hit compounds and analysed by the above assays. As shown in Figure 5A‐C, four of these compounds (#3, 18, 21, 22) proved to be intrinsically toxic and were removed from further analysis as they failed two of the three assays (#18, 21, 22) or displayed significant toxicity in the LDH release assay (#3).

Next, we re‐examined the potential of the remaining 14 compounds to reduce levels of Bax translocation in response to cisplatin over a concentration range of 100 nmol/L to 5 μmol/L. As shown in Figure 5D, five of the six 4σ compounds demonstrating significant Bax translocation suppression on retest with two of these (5 and 29) demonstrating significant Bax suppression at concentrations of 500 nmol/L or greater compared to controls. Thus, pharmacologic and toxicologic profiling indicated that compounds 5 and 29 warranted further investigation. Unblinding of test compound identifiers surprisingly revealed them to be forskolin and colforsin, both known to enhance the levels of cyclic AMP by promoting adenylyl cyclase activity. These findings prompted us to analyse a number of related compounds which act either directly or indirectly to modulate cAMP levels. These investigations resulted in the identification of one additional compound (rolipram) which was also observed to alter cisplatin‐mediated Bax translocation levels.

### 3.5 Identified small molecule inhibitors of Bax translocation reduce executioner caspase activity and enhance cell survival

**Figure 6:**
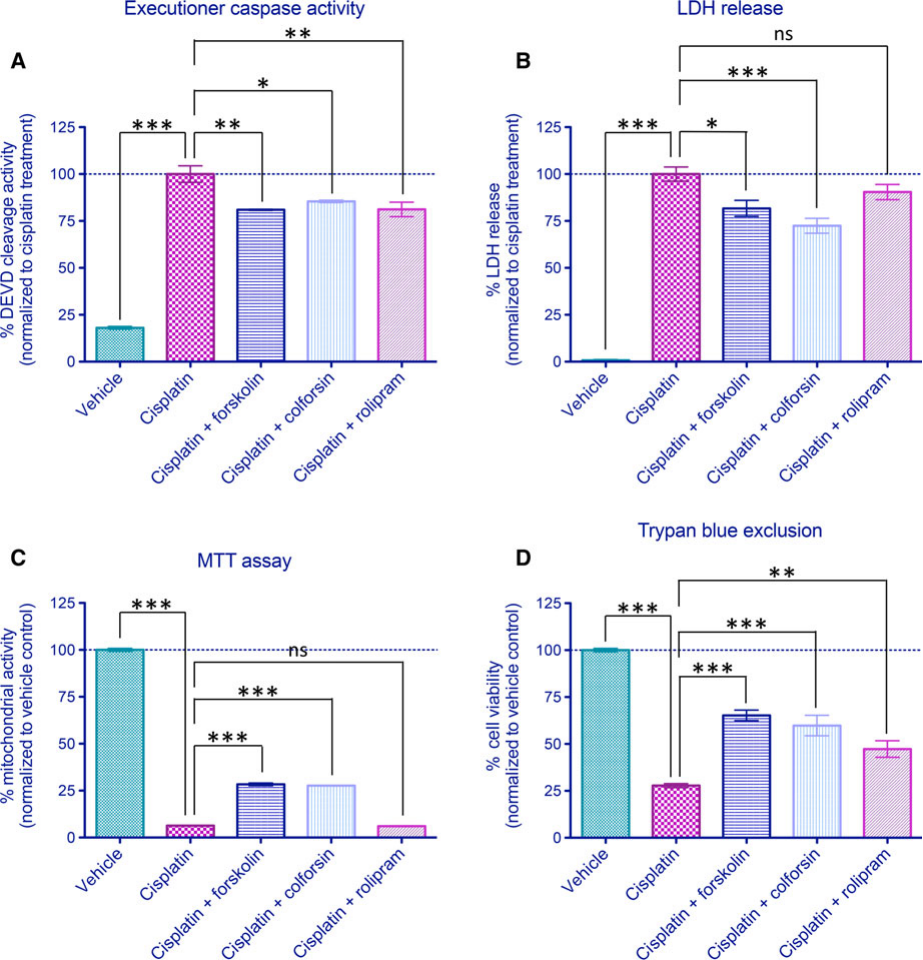
Cell death inhibition activity of compounds forskolin, colforsin and rolipram. Cells were exposed to cisplatin (50 μg/mL) for 8 h followed by washout with addition of vehicle or hit compounds (5 μmol/L) to test for efficacy in cell death inhibition. (A) Analysis of caspase‐3/7 (DEVD) activity in the presence of cisplatin. All three compounds identified significantly reduced levels of cisplatin‐induced executioner caspase activity. (B) Alterations of plasma membrane integrity as monitored by lactate dehydrogenase (LDH) release. Forskolin and colforsin significantly reduce the levels of LDH release compared to cisplatin controls, while rolipram exhibit a non‐significant trend toward reduced LDH release. (C) Analysis of mitochondrial activity as measured by MTT reveals significant increases by forskolin and colforsin but not rolipram. (D) Assessment of cell viability by trypan blue exclusion assay at 24 h following treatment. All three compounds exhibited significant protective effects against cisplatin‐mediated programmed cell death. Data are presented as normalized mean ± SEM for triplicate experiments for all treatment groups. *, **, and *** indicate statistical significance at *P* < 0.05, *P* < 0.01, and *P* < 0.001, respectively, by one‐way anova and Bonferroni's multiple comparison test between the indicated treatment groups. ns indicates not significant statistically

In order to assess the ability of forskolin, colforsin and rolipram to suppress the features of PCD downstream of Bax translocation, several viability assays were performed. To mirror the conditions of our high‐throughput screen, cells were exposed to cisplatin (50 μg/mL) for 8 hours prior to washout and treatment of either vehicle or hit compounds (5 μmol/L) for 24 hours before the assays. As shown in Figure 6A, executioner caspase activity (cleavage of fluorogenic DEVD substrate) was significantly inhibited following treatment with forskolin, colforsin or rolipram, consistent with a role for these compounds at or upstream of Bax translocation to inhibit the progression of apoptosis. We further examined the ability of these compounds to elicit cytoprotection by measuring LDH release (plasma membrane integrity) and MTT reduction (mitochondrial activity). As shown in Figure 6B, compounds forskolin and colforsin significantly reduced the levels of cisplatin‐induced LDH release, while rolipram exhibited a trend towards reductions in LDH release but did not achieve statistical significance. As shown in Figure 6C, MTT assay revealed modest enhancement of mitochondrial activity following the addition of forskolin or colforsin, but not rolipram, to cisplatin‐treated cells. Finally, protection against cisplatin‐induced cell death was assessed by trypan blue exclusion assay. All three compounds demonstrated significant rescue effects (Figure 6D), suggesting that manipulation of intracellular cAMP levels and reducing Bax translocation were indeed capable of protecting cells against cisplatin‐induced cell death.

**Figure 7:**
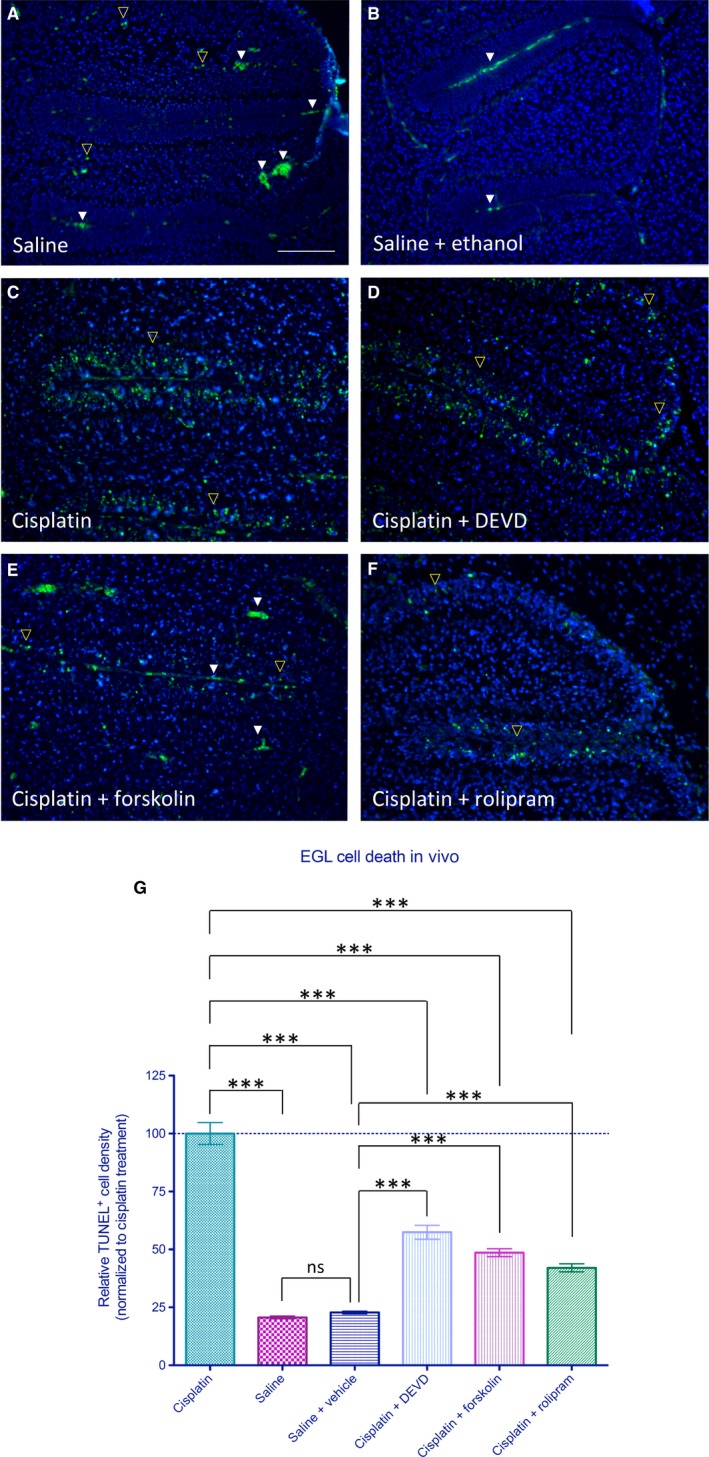
Pharmacologic inhibition of programmed cell death by identified compounds in vivo. Neuroprotective effects of hit compounds on cisplatin‐induced apoptotic cell death in vivo. (A) Level of TUNEL^+ ^early postnatal cell death observed following injection of saline control. Note that in addition to TUNEL^+ ^cells (yellow open arrowheads), fluorescence is also observed in red blood cells at this wavelength (identified by their biconcave shape—white arrowheads). (B) Level of cell death observed following injections of saline control and ethanol vehicle after 1 hour. (C‐D) Treatment with cisplatin resulted in a substantial rise in level of cell death within the external germinal layer (EGL) as observed by TUNEL, which was significantly reduced by DEVD pre‐treatment. (E‐F) Injection of forskolin at 1 hour following cisplatin treatment reduced the number of TUNEL^+ ^cells observed in the EGL as did similar treatment with rolipram. (G) Relative density of TUNEL^+ ^cells within the EGL as observed for each group quantified across four parasagittal sections with an inter‐section distance of 100 μm. Saline alone group showed no significant difference from saline + ethanol vehicle group. Data represent normalized means ± SEM, *n* = 6 animals per group. *** indicates statistical significance at *P* < 0.001 by one‐way anova and Bonferroni's multiple comparison test between the indicated treatment groups. ns indicates not significant statistically; scale bar: 100 μm

Previously, we have demonstrated that cerebellar granule cells display a profound dependence on caspase‐3 mediated apoptosis following NMDA blockade in the early postnatal period.^18^ In order to test the in vivo effectiveness of the identified compounds, we examined their potentials to protect cells of the EGL of the cerebellum from cisplatin‐induced caspase‐3‐dependent apoptosis (Figure 7A). As shown in Figure 7A and B, injection of compound vehicle induced no significant increase in EGL cell death. By contrast, injection of cisplatin at 7 mg/kg induced substantial EGL cell death (Figure 7C‐D) which was substantially diminished by the application of forskolin or rolipram 1 hour following cisplatin treatment (Figure 7E,F, respectively). Indeed, as quantified in Figure 7G, application of these agents at a concentration of 40 μmol/L at 1 hour following cisplatin treatment reduced levels of EGL cell death below that observed even following pre‐treatment with the caspase‐3/7 inhibitor z‐DEVD‐fmk 30 minutes before cisplatin treatment. This treatment reduced cisplatin‐mediated apoptotic cell death to 57% of levels observed in the cisplatin alone group. Consistent with our results observed in vitro*,* treatment with forskolin and rolipram at a similar in vivo concentration of 40 μmol/L reduced the levels of apoptotic cells to 49% and 42%, respectively. Taken together, our data suggest that these agents are capable of blocking more than caspase‐3/7‐mediated cell death in this system, consistent with their abilities to suppress of Bax translocation and consequentially reduce MOMP, which can trigger caspase‐independent cell death.

## 4 DISCUSSION

In the present study, we developed a high‐content high‐throughput system to examine the real‐time Bax translocation following PCD stimulation. We utilized CHO cell lines stably expressing EGFP‐Bax to screen for small molecule inhibitors of Bax translocation, a key and requisite step in the execution of mitochondrial‐mediated PCD. Examination of over 6000 compounds from four distinct chemical libraries resulted in the identification of 18 compounds which appeared to significantly reduce cisplatin‐induced Bax translocation. Further testing and analysis of these initial hits ultimately identified two chemically distinct pharmacologic agents which substantially reduced the levels of pro‐apoptotic Bax translocation, even when added 8 hours subsequent to the initiation of cell death as stimulated by cisplatin. Interestingly, uncoding of these agents revealed them to be two distinct modulators of cAMP signalling. Secondary screening of cAMP modulatory agents revealed that the phosphodiesterase inhibitor rolipram also provided significant inhibition of Bax translocation. To our knowledge, this is the first high‐throughput screen aimed at identifying small molecule inhibitors of Bax activation/translocation in order to inhibit the progression of PCD. The utility of this approach has been demonstrated previously in several high‐throughput screens performed to identify inhibitors of Bcl‐2 family proteins such as Bcl‐xL and Bcl‐B for use as anti‐cancer therapeutics.^21^, ^22^ For these studies, we incorporated several screening features aimed at enhancing the identification of clinically useful therapeutics. First, we have constructed our primary screen around a cell‐based system instead of an in vitro biochemical assay. Such systems select for compounds with appropriate membrane permeability and quickly filter out those possessing significant acute toxic effects. The use of dynamic core markers of apoptosis such as Bax translocation also allowed direct functional testing of compounds in an unbiased manner providing a means to identify any and all agents which influence the given process regardless of mechanism rather than narrowly focusing on a particular protein‐protein interaction for initial selection. We have chosen to examine the ability of small molecules to inhibit Bax translocation and enhance cell viability 8 hours *subsequent* to cisplatin‐induced cell injury in an attempt to identify compounds which do not act on early specific initiation events in PCD per se, but rather more generalized aspects of PCD progression with the aim of targeting a more clinically realistic therapeutic window. This approach stands in contrast to screening protocols in which functional inducer and small molecule therapeutic are tested coincident with one another, frequently identifying candidate compounds which ultimately fail in real clinical settings.^23^, ^24^ By taking a post‐injury approach for screening, it is hoped that the probability of cell‐based effects observed would translate into significant cellular rescue when applied in a physiologic context. As such, it is important to note that despite some compounds exhibiting relatively modest effects on Bax translocation in the high‐throughput screen, ultimately they were able to exert significant neuroprotective effects in vivo.

Screening for small molecule inhibitors of Bax translocation in response to cisplatin‐induced DNA damage identified two different modulators of cAMP signalling. Previously, several investigators have described protective effects resulting from elevated cAMP levels against p53‐mediated PCD.^25^, ^26^ However, the precise molecular mechanism underlying these observations remains unclear. Multiple pathways have been suggested, including modulation of p53 phosphorylation, altered expression of Bax and related pro‐apoptotic proteins such as Puma, induction of BDNF expression through its cAMP responsive element binding protein and direct alteration of Bax phosphorylation (inhibits its translocation to the mitochondria).^27^, ^28^, ^29^ In the present study, we show that the identified cAMP modulators significantly reduced the levels of downstream executioner caspase activity (Figure 7A). As we have only monitored the activation of Bax, cAMP‐independent Bax activation (eg direct interaction with p53) and Bax‐independent mechanisms (ie Bak‐mediated MOMP) could still have triggered cell death as illustrated by the relative minor protection against the loss of mitochondrial activity shown through MTT reduction assay (Figure 7C). Hence, in order to realistically rescue neurons damaged during acute neural injuries, pharmacologic modulation of multiple pathways are likely required.

In the present study, we describe the development of a robust high‐throughput screening approach to identify small molecule inhibitors of Bax translocation. Such screening resulted in the identification of two small molecules regulating cAMP signalling which significantly inhibit Bax translocation at sub‐micromolar concentrations. These findings demonstrate that pharmacologic manipulation of cAMP levels in vitro and in vivo alter the levels of neural apoptosis in the presence of cisplatin. However, these findings also highlight the complexities in modifying such injuries in vivo due to the regulated presence of multiple isoforms of PCD (eg necroptosis). As such, inhibition of several distinct PCD pathways may ultimately be required to provide effective therapeutic neuroprotective responses in vivo.

## CONFLICT OF INTEREST

The authors declare no conflicts of interest.
