# Supplementary figures and images for: Cell‐based high‐throughput screen for small molecule inhibitors of Bax translocation
Source: J Cell Mol Med. 2018 Dec 13;23(3):1784–97. doi: 10.1111/jcmm.14076 (PMC6378228; doi:10.1111/jcmm.14076)

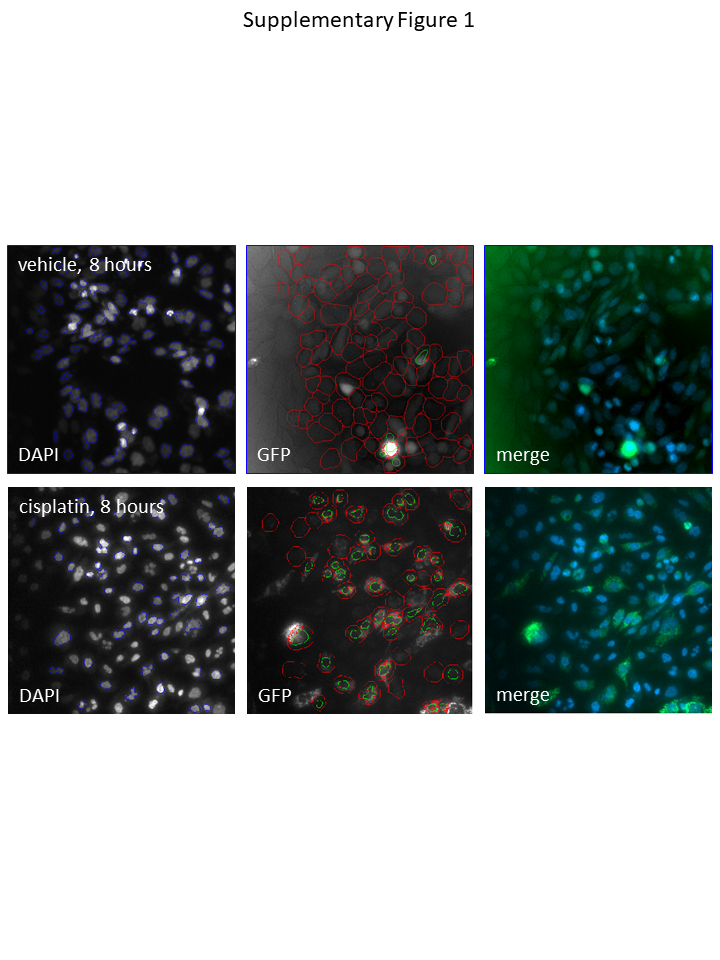

Supplement: Supplementary file 1 [file JCMM-23-1784-s001.TIF]
