# Supplementary material for: Cell‐based high‐throughput screen for small molecule inhibitors of Bax translocation
Source: J Cell Mol Med. 2018 Dec 13;23(3):1784–97. doi: 10.1111/jcmm.14076 (PMC6378228; doi:10.1111/jcmm.14076)
Supplement: Supplementary file 2 [file JCMM-23-1784-s002.docx]

**Supplementary Figure 1.** Analysis of Bax translocation by an optimized Spot Detection algorithm

Representative images of vehicle- and cisplatin-treated EGFP-Bax CHO cells were captured by Cellomics ArrayScan HCS and analyzed automatically by the modified Spot Detection algorithm. Nuclei were detected via the DAPI channel (left panels) to identify cells. Cells with nuclei on the edges of each captured image were excluded from analysis. A cellular profile was drawn for each cell based on a modifiable radius from the nuclear profile to identify the cell cytoplasm where green fluorescence signal representing EGFP-Bax would be detected. Dots were drawn by the algorithm based on the EGFP fluorescence signal, with cells that have ≥20 spots considered to have undergone Bax translocation (middle panels). Images in the right panels are merged fluorescence images from DAPI and EGFP channels.
